# Case Report: Immune checkpoint inhibitor–triggered anti-Ma2 paraneoplastic encephalitis in sarcomatoid pleural mesothelioma: a fatal case

**DOI:** 10.3389/fonc.2026.1739494

**Published:** 2026-03-09

**Authors:** Ilker Nihat Okten, Tuba Baydaş

**Affiliations:** Department of Medical Oncology, TC Sağlık Bakanlığı Göztepe Prof. Dr. Süleyman Yalçın City Hospital, Istanbul, Türkiye

**Keywords:** anti-Ma2 encephalitis, immune checkpoint inhibitors, neuroimmunology, paraneoplastic neurological syndrome, pleural mesothelioma

## Abstract

**Background:**

Neurological immune-related adverse events (N-irAEs) represent rare but potentially fatal complications of immune checkpoint inhibitor (ICI) therapy. Among these, encephalitis associated with paraneoplatic neuronal antibodies poses a major diagnostic and therapeutic challenge, as it blurs the distinction between drug-induced toxicity and tumor-driven autoimmunity.

**Case presentation:**

We report a 61-year-old male diagnosed with unresectable sarcomatoid pleural mesothelioma who was treated with first-line nivolumab plus ipilimumab. Following the third treatment cycle, the patient developed progressive hyperphagia, central sleep apnea, and autonomic dysfunction. Serum testing revealed strong positivity for anti-Ma2/Ta antibodies, while brain magnetic resonance imaging was unremarkable. Despite discontinuation of immunotherapy and treatment with high-dose corticosteroids and intravenous immunoglobulin, the patient experienced relentless neurological deterioration. Notably, follow-up positron emission tomography demonstrated complete metabolic tumor response. The patient ultimately died from progressive brainstem dysfunction.

**Discussion:**

Anti-Ma2–associated encephalitis is classically categorized as a paraneoplastic neurological syndrome mediated by cytotoxic T-cell responses against intracellular neuronal antigens. Recent evidence suggests that ICIs can unmask or accelerate latent paraneoplastic autoimmunity by amplifying pre-existing immune responses. In this context, our case is best interpreted as an ICI-triggered paraneoplastic neurological syndrome rather than a primary immune-related adverse event.

**Conclusion:**

This case highlights a fatal neurological complication occurring in parallel with complete oncologic remission, underscoring the paradox of effective cancer immunotherapy precipitating catastrophic immune-mediated neurotoxicity. Early recognition of prodromal neurological symptoms and heightened awareness of paraneoplastic syndromes in the ICI era are critical to improving patient outcomes.

## Introduction

Immune checkpoint inhibitors (ICIs) have transformed the therapeutic landscape of oncology, leading to durable survival benefits across a wide range of solid malignancies. By releasing inhibitory signals on cytotoxic T lymphocytes, agents targeting programmed death-1 (PD-1), programmed death-ligand 1 (PD-L1), and cytotoxic T-lymphocyte–associated protein 4 (CTLA-4) restore antitumor immune surveillance and enable long-term disease control in a subset of patients (1). However, this immune activation is inherently non-selective and can result in immune-related adverse events (irAEs) affecting virtually any organ system (2).

While gastrointestinal, endocrine, pulmonary, and cutaneous toxicities are most frequently encountered, neurological immune-related adverse events (N-irAEs) represent a rare but clinically significant complication of ICI therapy. N-irAEs occur in approximately 1–4% of patients receiving ICI monotherapy and up to 12–14% in combination regimens, and are associated with substantial morbidity and mortality (3).

Among central nervous system toxicities, encephalitis represents the most severe and potentially fatal phenotype. ICI-associated encephalitis may present as limbic, brainstem, or diffuse encephalopathy, often accompanied by cognitive dysfunction, seizures, autonomic instability, or sleep disorders. Importantly, a subset of these cases is associated with paraneoplastic neuronal antibodies, most notably anti-Hu and anti-Ma2, blurring the classical distinction between drug-induced toxicity and tumor-driven autoimmunity (3).

Anti-Ma2–associated encephalitis belongs to the group of paraneoplastic neurological syndromes (PNS) mediated by immune responses against intracellular neuronal antigens. These disorders are predominantly driven by cytotoxic CD8^+^ T-cell infiltration of the central nervous system and are characterized by poor responsiveness to immunotherapy and frequent irreversible neurological damage (4). Recent evidence suggests that immune checkpoint blockade can unmask or accelerate latent paraneoplastic autoimmunity, rather than merely induce *de novo* immune toxicity (3).

Pleural mesothelioma represents a biologically aggressive malignancy with limited therapeutic options. The sarcomatoid subtype, in particular, is associated with profound chemoresistance and extremely poor prognosis. The CheckMate-743 trial established nivolumab plus ipilimumab as standard first-line therapy in unresectable non-epithelioid mesothelioma, leading to widespread adoption of dual immune checkpoint blockade in this population (5).

Here, we report a fatal case of anti-Ma2–associated brainstem encephalitis in a patient with sarcomatoid pleural mesothelioma treated with nivolumab and ipilimumab, illustrating the complex interplay between tumor immunogenicity, immune checkpoint blockade, and catastrophic paraneoplastic neurotoxicity.

## Case presentation

A 61-year-old male with no known neurological history presented in February 2024 with progressive right shoulder pain and dyspnea. Thoracic computed tomography revealed right-sided pleural effusion with diffuse pleural thickening with direct diaphragmatic invasion. Video-assisted thoracoscopic surgery (VATS) biopsy demonstrated sarcomatoid pleural mesothelioma, composed of pleomorphic spindle cells with high mitotic activity (24/2 mm²), focal necrosis, and diffuse infiltrative growth pattern. Immunohistochemistry was positive for calretinin, GATA3, and cytokeratin AE1/AE3, and negative for TTF-1 and p40, confirming the diagnosis. Based on imaging and clinical evaluation, the disease was considered unresectable. According to the 8th edition IASLC TNM staging system for pleural mesothelioma, the disease was staged as *cT4N0M0 (Stage IIIB)* due to diffuse involvement of the right pleura with direct diaphragmatic invasion, rendering the tumor unresectable.

The patient was started on first-line immune checkpoint blockade according to the CheckMate-743 protocol:

- 15 March 2024: Nivolumab + ipilimumab (Cycle 1)
- 29 March 2024: Nivolumab (Cycle 2)
- 15 April 2024: Nivolumab (Cycle 3)
- 3 May 2024: Nivolumab + ipilimumab (Cycle 4)
- 17 May 2024: Nivolumab (Cycle 5)
- 31 May 2024: Nivolumab (Cycle 6)

After the third treatment cycle, the patient’s general condition improved and appetite increased. During the fourth cycle, he began to report loud nocturnal snoring. Following the fifth cycle, he developed excessive daytime somnolence. After the sixth cycle, the patient experienced rapid-onset hyperphagia and weight gain, accompanied by episodes of central sleep apnea; despite these symptoms, his Eastern Cooperative Oncology Group performance status remained 0, and he subjectively reported feeling well. Four days later, on 4 June, he developed acute urinary retention and autonomic dysfunction, prompting urgent neurological evaluation and subsequent admission to the intensive care unit due to prolonged episodes of sleep apnea.

Brain magnetic resonance imaging performed on 4 June 2024 showed no evidence of metastasis, infarction, or structural abnormality. Given the constellation of hypothalamic, brainstem, and autonomic symptoms, a comprehensive autoimmune and paraneoplastic evaluation was performed.

Serum testing using a commercial immunoblot panel revealed strong positivity for anti-Ma2/Ta antibodies (3+), while all other paraneoplastic antibodies were negative, including anti-Hu, anti-Yo, anti-Ri, CRMP5, amphiphysin, recoverin, SOX1, ZIC4, GAD65, and anti-titin. In addition, a limbic encephalitis panel was negative for NMDA-R, AMPA1/2, GABA-B, CASPR2, LGI1, and DPPX antibodies. Broad ganglioside panels were also negative. The assay was performed on serum using a commercial immunoblot method; cerebrospinal fluid analysis was not available at the time of testing.

Infectious etiologies were excluded through negative viral serologies and absence of systemic inflammatory markers. There was no metabolic, toxic, or structural explanation for the rapidly progressive neurological syndrome. Taken together, the clinical phenotype and serological profile supported a diagnosis of anti-Ma2–associated autoimmune encephalitis.

Immune checkpoint therapy was permanently discontinued after cycle six. The patient received oral corticosteroids (2 mg/kg/day) without clinical improvement, followed by three-day intravenous methylprednisolone pulse therapy and intravenous immunoglobulin (IVIG, total dose 45 g), which produced only transient symptomatic benefit. Maintenance therapy with prednisone 10 mg/day and monthly IVIG was continued for three months.

**Figure 1:**
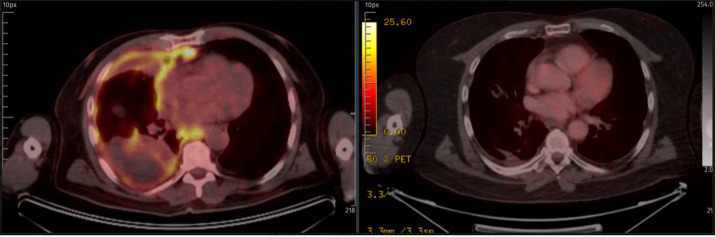
Baseline and post-treatment PET-CT showing complete metabolic response.

Despite ongoing neurological deterioration, a follow-up 18F-FDG PET-CT on 16 August 2024 demonstrated complete metabolic response of the pleural disease (**Figure 1**). Nevertheless, the patient developed recurrent episodes of central apnea requiring intensive care admission and died on 16 February 2025 due to progressive brainstem dysfunction. At the time of death, there was no radiological or clinical evidence of active malignancy (**Table 1**).

**Table 1:** Timeline of clinical events, immunotherapy cycles, symptom onset, and interventions.

Date	Event
February 15, 2024	Diagnosis of sarcomatoid pleural mesothelioma.
March 15, 2024 (Cycle 1)	Nivolumab + ipilimumab initiated according to the CheckMate-743 protocol.
March 29, 2024 (Cycle 2)	Nivolumab.
April 15, 2024 (Cycle 3)	Nivolumab – improvement in general condition and increased appetite.
May 3, 2024 (Cycle 4)	Nivolumab + ipilimumab – onset of loud nocturnal snoring.
May 17, 2024 (Cycle 5)	Nivolumab – development of excessive daytime somnolence.
May 31, 2024 (Cycle 6)	Nivolumab – rapid-onset hyperphagia and weight gain; episodes of central sleep apnea; ECOG PS 0.
June 4, 2024	Acute urinary retention and autonomic dysfunction; neurological evaluation and ICU admission due to prolonged sleep apnea. Brain MRI showed no metastasis, infarction, or structural abnormality.
August 16, 2024	FDG PET-CT demonstrated complete metabolic response.
February 16, 2025	Death due to immune-mediated encephalitis.

## Discussion

Anti-Ma2–associated encephalitis belongs to the spectrum of autoimmune neurological disorders characterized by immune responses against intracellular neuronal antigens, classically categorized as paraneoplastic neurological syndromes (PNS) (6). Unlike surface antibody–mediated encephalitides (e.g., NMDA-R, LGI1), which are primarily antibody-driven and often reversible, Ma2-associated disease is predominantly mediated by cytotoxic T-cell responses directed against neurons expressing tumor-shared antigens (7).

Historically, anti-Ma2 encephalitis has been described in association with testicular cancer, lung cancer, and less frequently with other solid tumors (8). However, recent large cohort studies have challenged the traditional paradigm that anti-Ma2 is strictly paraneoplastic. In a nationwide French cohort, Vaisvilas et al. demonstrated that up to one-third of patients with anti-Ma2–associated neurological syndromes had no detectable malignancy despite long-term follow-up, indicating that Ma2 autoimmunity represents a broader autoimmune encephalitis spectrum rather than an exclusively tumor-driven entity (9).

A recent case reported by Tada et al. described anti-Ma2–associated paraneoplastic neurological syndrome in a patient with sarcomatoid pleural mesothelioma in the absence of immune checkpoint therapy, demonstrating that this tumor histology itself may express Ma2 antigens and predispose to paraneoplastic autoimmunity (10).

The widespread adoption of immune checkpoint inhibitors (ICIs) has fundamentally altered the epidemiology of autoimmune neurological syndromes. Vogrig et al. reported a more than twofold increase in the frequency of anti-Ma2 encephalitis following the introduction of ICIs, with cases observed across multiple tumor types, including pleural mesothelioma (3). Importantly, in several patients, anti-Ma2 antibodies were detectable prior to ICI exposure, suggesting that immune checkpoint blockade does not induce *de novo* autoimmunity but rather amplifies pre-existing immune responses by removing physiological immune tolerance mechanisms.

This phenomenon supports the unmasking model, in which ICIs act as immune accelerators in immunologically primed individuals, facilitating expansion of autoreactive CD8^+^ T cells directed against intracellular neuronal antigens shared with tumor tissue (11). In this context, immune checkpoint blockade should not be interpreted as the primary etiologic agent but rather as a catalyst that unleashes latent paraneoplastic autoimmunity. Our case fits this paradigm: neurologic symptoms emerged after immune activation, progressed despite tumor eradication, and culminated in fatal brainstem dysfunction.

From a mechanistic standpoint, anti-Ma2 encephalitis is characterized by infiltration of cytotoxic CD8^+^ T lymphocytes into affected brain regions, particularly the limbic system, diencephalon, and brainstem (4, 7). Neuropathological studies have demonstrated neuronal loss with prominent T-cell infiltration and minimal antibody deposition, underscoring the limited role of circulating antibodies in disease pathogenesis. This explains why Ma2-associated encephalitis responds poorly to conventional antibody-targeted therapies and carries a substantially worse prognosis than surface antibody–mediated autoimmune encephalitides (12).

Diagnostic delay represents a critical factor contributing to adverse outcomes in Ma2-associated disease. Early symptoms are often nonspecific and include sleep disturbance, hyperphagia, behavioral changes, or autonomic dysfunction, which may be misattributed to metabolic, psychiatric, or treatment-related causes. In our patient, subtle prodromal manifestations including sleep-disordered breathing and excessive daytime somnolence emerged during ongoing immune checkpoint therapy, while more overt neurological deterioration rapidly evolved after the sixth treatment cycle. In retrospect, these early features were consistent with diencephalic and brainstem involvement, which are increasingly recognized as early presentations of anti-Ma2–associated encephalitis. This temporal pattern highlights the inherent diagnostic challenge of distinguishing evolving paraneoplastic neurological syndromes from nonspecific treatment-related symptoms in the setting of active immunotherapy.

The limited efficacy of immunosuppressive therapy in this case is biologically consistent with the underlying CD8^+^ T-cell–mediated pathology. High-dose corticosteroids and intravenous immunoglobulin primarily target humoral immune mechanisms and are often insufficient in intracellular antigen–mediated syndromes (13). Escalation to plasmapheresis, rituximab, or cyclophosphamide may be required to suppress cellular immunity, yet even aggressive immunosuppression frequently fails to reverse established neuronal damage in Ma2-associated disease (14). In Vogrig’s cohort, mortality exceeded 50% despite multimodal immunotherapy, highlighting the intrinsically refractory nature of this condition (3).

Our case also illustrates a paradox increasingly encountered in modern oncology: profound antitumor immune activation occurring simultaneously with catastrophic immune-mediated neurotoxicity. The patient achieved complete metabolic remission of pleural mesothelioma while succumbing to an autoimmune neurological complication triggered by the same immune response responsible for tumor control. This duality underscores the ethical and clinical challenge of immune checkpoint therapy in patients susceptible to immune-mediated neurological syndromes.

In conclusion, this case supports the emerging concept that anti-Ma2–associated encephalitis in the era of immunotherapy should be regarded as an ICI-triggered paraneoplastic neurological syndrome rather than a primary immune-related adverse event. Early recognition of prodromal neurological symptoms, understanding of intracellular antibody biology, and rapid escalation of cellular immunosuppression are essential to improve outcomes in this devastating condition.

## Learning points

1. Neurological symptoms emerging during immune checkpoint inhibitor therapy should not be automatically classified as immune-related adverse events; the presence of intracellular neuronal antibodies (e.g., anti-Ma2) strongly suggests an underlying paraneoplastic neurological syndrome.
2. Anti-Ma2–associated encephalitis is predominantly mediated by cytotoxic T-cell mechanisms and is frequently refractory to standard antibody-directed immunotherapies, resulting in poor neurological outcomes despite aggressive treatment.
3. Immune checkpoint inhibitors may act as immune amplifiers, unmasking or accelerating latent paraneoplastic autoimmunity rather than inducing *de novo* neurological toxicity.
4. Prodromal manifestations such as sleep disturbances, hyperphagia, and autonomic dysfunction should prompt early neurological evaluation in patients receiving ICIs, as delayed recognition may lead to irreversible neurological injury.
5. Successful oncologic response to immunotherapy does not preclude fatal immune-mediated neurotoxicity, underscoring the ethical and clinical complexity of immune checkpoint therapy in patients susceptible to paraneoplastic syndromes.

## Data Availability

The raw data supporting the conclusions of this article will be made available by the authors, without undue reservation.
